# Monodispersed ZnO Nanoparticles and Their Use in Heterojunction Solar Cell

**DOI:** 10.1155/2013/260521

**Published:** 2013-11-10

**Authors:** Dinesh Patidar, Anusaiya Kaswan, N. S. Saxena, Kananbala Sharma

**Affiliations:** Semi-Conductor and Polymer Science Laboratory, Department of Physics, University of Rajasthan, Jaipur 302004, India

## Abstract

Monodispersed ZnO nanoparticles have been synthesised in ethylene glycol medium using zinc acetate and sodium hydroxide at room temperature through ultrasonic treatment. The monodispersed ZnO nanoparticles were characterized by XRD, TEM, SEM, and optical spectroscopy. The results indicate that ZnO shows the hexagonal wurtzite structure having 8 nm average particle size with the band gap of 3.93 eV. ZnO nanoparticles blended with P3HT show the improvement in the interchains and intrachains ordering as compared to pure P3HT. The power conversion efficiency of P3HT/ZnO solar cell is found to be 0.88%, which is comparable with the result obtained by other researchers.

## 1. Introduction

Semiconducting nanocrystalline materials have been intensively investigated because of their fundamental scientific interest and many technological applications [1–3]. Among them, zinc oxide (ZnO) is an important basic material from a versatile application point of view due to its large band gap (3.37 eV), low cost, and luminescent properties [4]. It is widely used in many fields, such as UV lasers [5], catalyst [6], gas sensors [7, 8], transparent conductive oxide [9], varistors [10], and light emitting diodes [11, 12]. 

Besides these applications, ZnO is also a very attractive semiconductor for use in solar cells due to its abundance and low cost, nontoxicity, high electron mobility, low crystallization temperature, large exciton binding energy (60 meV), and easy synthesis. Recently, it is observed that ZnO can be used as electron acceptor to dissociate excitons produced in conjugated polymers. This combines the advantages of both organic semiconductors (flexibility, solutions processing) and inorganic semiconductors (stability, high mobility). This type of hybrid organic-inorganic solar cells can be fabricated by blending ZnO nanoparticles and conjugated polymer (bulk heterojunction: BHJ) and also by impregnation of conjugated polymer on nanostructured ZnO, which is coated on indium tin oxide (ITO). First successful attempt was made to fabricate P3HT/ZnO nanorods photovoltaic cell by replacing TiO_2_ [13]. In this case, a promising performance of the device based on ZnO nanorods was obtained, which is due to the ease of electron transport and collection. Ravirajan et al. [14] have reported the effect of nanoparticles morphology and interfacial modification on the performance of P3HT/ZnO photovoltaic device. They found that photovoltaic device based on nanorod structure treated with amphiphilic dye before deposition of P3HT polymer yields four times greater power conversion efficiency as compared to untreated device. The 1.6%, and 0.9% power conversion efficiencies have been observed by blending ZnO nanoparticles with MEH-PPV [15] and P3HT, respectively. Olson et al. [16] have studied ZnO nanofiber electrodes impregnated with P3HT and they obtained 0.53% efficiency in ITO/ZnO/P3HT/Ag devices. Peiró et al. [17] have observed 0.2% efficient ITO/ZnO/P3HT/Au devices after coating ZnO with a dye. Electrons are extracted from the ZnO conduction band (4.35 eV) by ITO, while holes are extracted from the 5.2 eV P3HT HOMO level by a high work function top contact such as Au or Ag in the BHJ configuration. It is noticed that the power conversion efficiency of the device based on ZnO nanorod arrays is even lower than that based on nanoparticles, which is partially due to the smaller interface area in the former devices. It is believed that the enhanced performance of device comes from the increased heterojunction area. The charge separation and transport in the device strongly depend on the morphology and orientation of the nanoparticles. 

Therefore, the synthesis of monodispersed ZnO nanocrystals is a very important aspect from the point of view of its solar cell's applications because its properties depend strongly on their dimensions [18] and morphology. It has a strong tendency to agglomerate and at last become big conglomerations, which influence the efficiency of solar cells. Various methods have been developed for synthesizing low-dimensional ZnO nanostructures such as solvothermal method [19], hydrothermal method [20, 21], physical vapor deposition (PVD) [22], thermal decomposition [23], chemical vapor deposition (CVD) [24], metal-organic vapor-phase epitaxy [25], metal-organic CVD [26], template-assisted growth [27], sputtering [28], and various solution methods [29–33]. Recently, the synthesis of ZnO nanorods using an alcohol thermal process was reported [34]. In this work, we report a simple, inexpensive, and slightly modified alcohol thermal process for synthesis of monodispersed ZnO nanoparticles. In this process, monodispersed ZnO nanoparticles have been prepared at room temperature in place of low temperature condition using ultrasonic treatment and characterized through the X-ray diffraction (XRD), transmission electron microscope (TEM), scanning electron microscope (SEM), and optical spectroscopy. The prepared monodispersed ZnO nanoparticles have been used to fabricate P3HT/ZnO solar cell, which was characterized through the optical spectroscopy and electrical measurements.

## 2. Experimental Details

Zn(Ac)_2_·2H_2_O, NaOH, ethylene glycol, and oleic acid have been purchased from Merk. ITO and P3HT have been procured from Sigma-Aldrich. All the reagents are of analytical grade and were used without further purification.

### 2.1. Synthesis of ZnO Nanoparticles

ZnO nanoparticles were prepared by an improved method derived from Spanhel and Anderson [35], Qian et al. [36], and Ge et al. [37]. A typical experiment to synthesize 8 nm ZnO nanoparticles is as follows. 0.04 M of Zn(Ac)_2_·2H_2_O was dissolved in 100 mL of absolute ethylene glycol under magnetic stirring at 80°C and 0.04 M of NaOH was also dissolved in 100 mL of absolute ethylene glycol using magnetic stirring for 20 min. Then, ethylene glycol solution containing NaOH was dropped into ethylene glycol solution containing Zn(Ac)_2_·2H_2_O at room temperature under strong magnetic stirring for 3 hrs. The mixture solution was treated ultrasonically for 30 mins. Then, 0.50 mL oleic acid as surfactant was added into the solution to avoid agglomeration of these nanoparticles. After this, monodispersed ZnO particles were obtained by centrifugation, throwing away the supernatant layer and the solid product was washed with ethanol, acetone, and deionized water sequentially. Then, monodispersed ZnO nanoparticles were dried in vacuum at room temperature.

### 2.2. Fabrication of Photovoltaic Devices

First of all, 10 mg/mL of P3HT was dissolved in chloroform by magnetic stirrer. Ten mg/mL of the above-obtained ZnO nanoparticles was also dissolved in chloroform. Chloroform containing ZnO was sonicated by ultrasonication for 1 hr to get the uniform dispersion of ZnO nanoparticles in chloroform. When the P3HT was completely dissolved in chloroform then 50 wt.% of the ZnO was added in the chloroform solution containing P3HT. This solution was thoroughly stirred for 24 hrs. Then prepared solution was again sonicated by ultrasonicator for one hour to get the uniform dispersion of ZnO nanoparticles in P3HT solution. After this, the film of P3HT/ZnO composite has been deposited by spin coating on ITO, which was cleaned several times with methanol, acetone, and deionized water, at 2000 rpm for 1 minute and 4000 rpm for 1 minute consequently to modify the surface of device. The Al electrode was then deposited on the top of P3HT/ZnO film at high vacuum. So prepared P3HT/ZnO solar cell was annealed at 100°C temperatures for a period of 5 minutes under vacuum. After 5 minutes, the device was left in the vacuum in order to cool down to room temperature and then the measurements were performed. 

### 2.3. Characterization Techniques

XRD measurement was performed with Philips X'pert X-ray diffractometer at a scanning rate of 3° per minute between 10 and 70°. The source used for this study was Cu, K*α* (*λ* = 1.5406 Å)  operated at 40 mA and 45 kV.

TEM measurement was performed on Tecnai G2 30 U-Twin system operating at an accelerating voltage of 200 kV. The sample for TEM measurement was prepared by dispersing the ZnO nanoparticles in chloroform using ultrasonicator. A drop of prepared solution was placed on the carbon coated copper grid and the solvent was removed by evaporation at room temperature before the TEM measurement.

SEM image of ZnO nanoparticles in powder form was obtained on Carl Zeiss EVO 18 SEM system with an accelerating voltage of 20 kV. 

The optical absorption spectra of ZnO nanoparticles, P3HT, and P3HT/ZnO solar cell were recorded over the wavelength range from 300 to 800 nm using Shimadzu spectrophotometer model 1800. 

Current-voltage (*I*-*V*) measurement was taken in air at room temperature using Keithley electrometer 6517 A. For photovoltaic characterization, the P3HT/ZnO solar cell was illuminated with 10 mW/cm^2^ white light illumination from a halogen lamp through the glass/ITO side. The calculation of the power conversion efficiency (*η*) has been performed using the equation

(1) $$\begin{array}{c} \eta =\frac{V_{\text{oc}}I_{\text{sc}}\text{FF}}{P_{\text{in}}}, \end{array}$$

where *V*_oc_, *I*_sc_, FF, and *P*_in_ are the open circuit voltage, the short circuit current density, the fill factor, and power of the incident light. 

The fill factor (FF) measures the quality of the solar cell as a power source and is defined as the ratio between the maximum power delivered to an external circuit and the potential power according to

(2) $$\begin{array}{c} \text{FF}=\frac{V_{\max}I_{\max}}{V_{\text{oc}}I_{\text{sc}}}, \end{array}$$

where *V*_max⁡_ and *I*_max⁡_ are the values of the current density and voltage for maximizing the product of the  *I*-*V*  curve in the fourth quadrant, where the device operates as an electrical power source.

## 3. Results and Discussion

**Figure 1:**
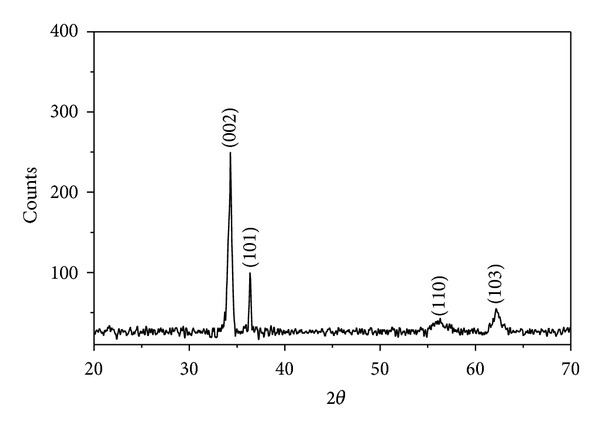
XRD of monodispersed ZnO nanoparticles.

Figure 1 shows the XRD pattern of ZnO, synthesized in alcohol medium. The peaks observed at 2*θ* = 34.03°, 36.35°, 56.36°, and 62.25° corresponding to the lattice planes (002), (101), (110), and (103), respectively, are indicative of wurtzite hexagonal structure of ZnO. All the peaks are matched with standard JCPDS card no. 361451. It seems that these ZnO particles possess a high crystallinity, since all the peaks are very sharp. No additional peaks of impurities were detected in the XRD pattern indicating the high purity of prepared nanoparticles.

**Figure 2:**
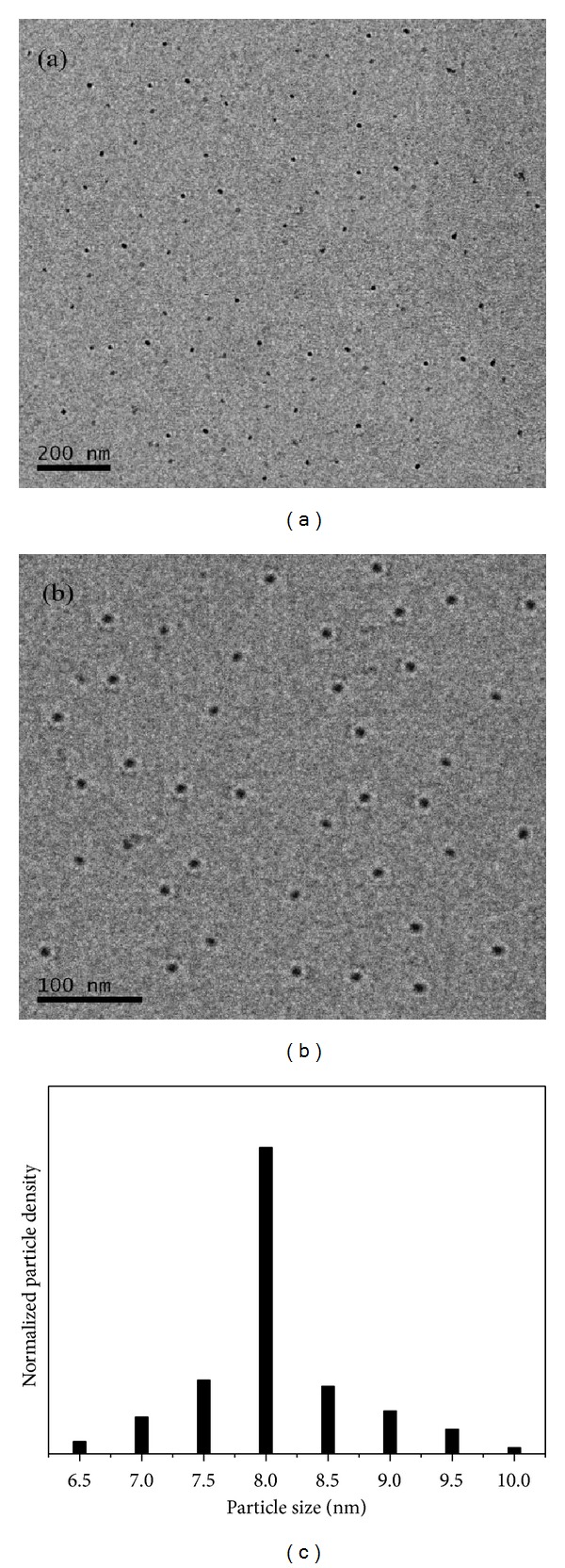
TEM images of ZnO nanoparticles at (a) 200 nm scale bar, (b) 100 nm scale bar, and (c) size distribution of ZnO nanoparticles.

**Figure 3:**
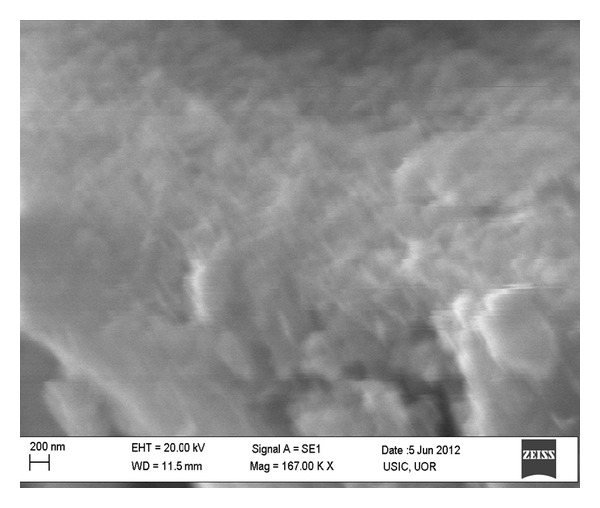
SEM images of ZnO nanoparticles.

The morphology of ZnO nanoparticles was investigated by TEM and SEM. The TEM images are shown in Figures 2(a) and 2(b) at 200 and 100 nm scale bar, respectively. The structure and shape of the particles are measured from the TEM measurement. The shape of particles is spherical as observed from TEM image. It is observed that the particles are well separated and distinguishable from each other. The obtained ZnO nanoparticles are monodispersed with 8 nm average particle size and size distribution of ZnO nanoparticles is shown in Figure 2(c).  Figure 3 shows the SEM image of ZnO nanoparticles. The image also depicts the nearly spherical morphology of the particles.

**Figure 4:**
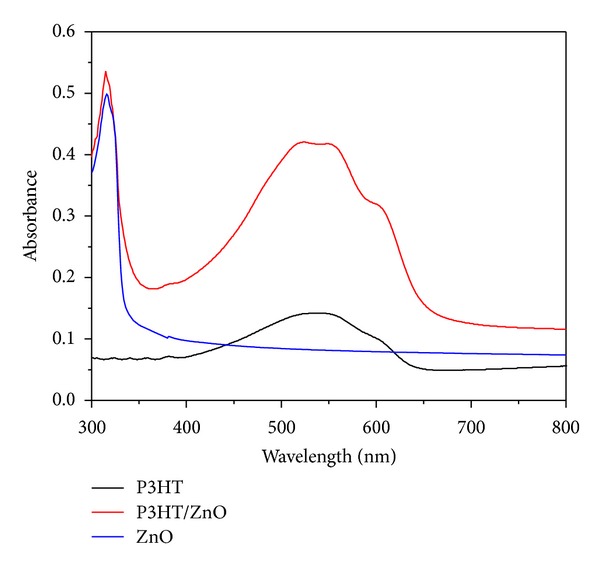
Absorption spectra of ZnO nanoparticles, P3HT, and P3HT/ZnO solar cell.

Figure 4 shows the absorption spectra of pure P3HT, ZnO nanoparticles, and 50 : 50 wt.% P3HT/ZnO solar cells annealed at 100°C for 5 minute in vacuum. In the absorption spectrum of pure P3HT, the original absorption of P3HT centred at 550 nm and 512 nm corresponds to *π*-π* transition, which is due to the 0-0 and the 0-1 transitions, respectively, where 0–*n* means a vibronic transition from the ground vibrational state of the ground singlet electronic state to the *n*th vibrational state of the first excited singlet electronic state and is in very good agreement with values reported in the literature [38, 39]. The transitions (0-0) and (0-1) are associated with the interchain and intrachain packing orders, respectively [40]. A peak corresponding to 315 nm (3.93 eV) is observed in absorption spectrum of monodispersed ZnO nanoparticles, which indicates the blue shifting of absorption peak as compared to its bulk counterpart. This confirms the nanostructure of the prepared ZnO. 

As the 50 wt.% ZnO nanoparticles are added in P3HT, an increment in the relative intensity of absorption is observed and sharp shoulder at 600 nm is also observed in Figure 4, which is also due to P3HT [39]. It is clear that the insertion of ZnO nanoparticles modify the absorption spectrum. This means that polymer conformation is strongly modified by its interaction with ZnO nanoparticles. The incorporation of ZnO nanoparticles modifies the polymer structure promoting polymer ordering (higher crystallinity) [41]. Moreover, this order increases interchain interactions in composite of P3HT and ZnO nanoparticles compared to the pure polymer. The shoulder at 600 nm (2.02 eV) represents a transition of a different nature. Brown et al. [38] showed compelling evidence that this transition can be attributed to an interchain absorption, corresponding to the formation of an exciton delocalized over multiple P3HT chains, the intensity of which is correlated with the degree of (torsional) order in the polymer. The presence of this shoulder indicates the enhanced hole mobility in the polymer [42].

**Figure 5:**
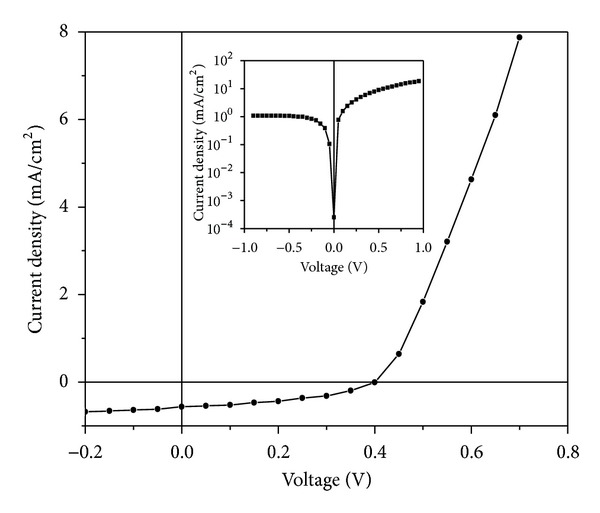
Current density-voltage characteristic of P3HT/ZnO solar cell under illumination and inset under dark.

In order to make use of monodispersed ZnO nanoparticles in solar cell application, ZnO nanoparticles have been blended with P3HT taking 50 : 50 wt.%. The current density-voltage (*J*-*V*) characteristic curve has been recorded using Keithley electrometer under the illumination intensity of 10 mW/cm^2^. The obtained result is plotted in Figure 5. Inset in Figure 5 shows the  *J*-*V*  characteristic curve in dark. The cell parameters of the device such as power conversion efficiency, open circuit voltage, short circuit current density, and fill factor have been determined using the  *J*-*V*  characteristic curve under illumination condition. The photovoltaic performance of the solar cell shows a short circuit current density of 0.56 mA/cm^2^, an open circuit voltage of 0.40 V, and fill factor of 0.40. The power conversion efficiency of 0.88% is obtained for P3HT/ZnO solar cell, which is comparable with the result reported by other workers [43]. 

## 4. Conclusions

The following conclusions have been drawn from the above studies.

1. Monodispersed ZnO nanoparticles of hexagonal wurtzite structure with an average size of 8 nm were synthesized at room temperature by ultrasonic treatment using ethylene glycol as reaction medium and zinc acetate and sodium hydroxide as reactants.
2. A film of P3HT/ZnO composite deposited by spin coating can serve as a solar cell.
3. An increase in the intensity of absorption spectrum of P3HT/ZnO as compared to P3HT indicates the improvement of interchain and intrachain ordering of P3HT with the addition of ZnO.
4. The power conversion efficiency of fabricated solar cell is comparable with the efficiency of other solar cells reported in the literature.
